# 免疫抑制治疗后三个月未获血液学反应的重型/极重型再生障碍性贫血患者六个月疗效评估

**DOI:** 10.3760/cma.j.issn.0253-2727.2022.05.008

**Published:** 2022-05

**Authors:** 向荣 胡, 馨 赵, 莉 张, 丽萍 井, 文睿 杨, 园 李, 蕾 叶, 康 周, 建平 李, 广新 彭, 慧慧 樊, 洋 李, 洋 杨, 佑祯 熊, 凤奎 张

**Affiliations:** 中国医学科学院血液病医院（中国医学科学院血液学研究所），实验血液学国家重点实验室，国家血液系统疾病临床医学研究中心，贫血诊疗中心，天津 300020 State Key Laboratory of Experimental Hematology, National Clinical Research Center for Blood Diseases, Institute of Hematology & Blood Diseases Hospital, Chinese Academy of Medical Sciences & Peking Union Medical College, Tianjin 300020, China

**Keywords:** 贫血，再生障碍性, 免疫抑制治疗, 预后因素, Anemia, aplastic, Immunosuppressive therapy, Prognostic factors

## Abstract

**目的:**

再评估影响免疫抑制治疗（IST）后3个月未获血液学反应的重型/极重型再生障碍性贫血（SAA/VSAA）患者6个月疗效的因素。

**方法:**

回顾性分析2017−2018 年连续收治的173例初治行IST且治疗后3个月未获血液学反应的SAA/VSAA患者的临床资料，对IST后3个月时的临床特征和血液学参数进行再评估，通过单因素和多因素分析找出影响6个月疗效获得的相关指标。

**结果:**

单因素分析结果显示IST后3个月无效患者的HGB（*P*＝0.017）、PLT（*P*＝0.005）、网织红细胞绝对计数（ARC）（*P*<0.001）、环孢素A血药浓度谷值（CsA-C0）（*P*＝0.042）、血清可溶性转铁蛋白受体（sTfR）（*P*＝0.003）、网织红细胞绝对计数改善值（ARC^Δ^）（*P*<0.001）、血清可溶性转铁蛋白受体改善值（sTfR^Δ^）（*P*<0.001）与IST后6个月疗效有关。多因素分析结果显示PLT<10×10^9^/L（*P*＝0.020）和ARC^Δ^<6.9×10^9^/L（*P*<0.001）是IST后3个月未获血液学反应患者6个月疗效的危险因素。IST后6个月未获血液学反应组3年总生存率［（80.1±3.9）％对（97.6±2.6）％，*P*＝0.002］和无事件生存率［（31.4±4.5）％对（86.5±5.3）％，*P*<0.001］均明显低于获得血液学反应组。

**结论:**

对IST后3个月未获血液学反应的SAA/VSAA患者再评估以预测其6个月疗效非常重要；IST后3个月残存造血仍是影响预后的主要参数；ARC^Δ^可反应骨髓造血是否正在恢复及恢复的程度；IST后3个月无效患者若ARC^Δ^<6.9×10^9^/L，无论PLT为何值，IST后6个月的血液学反应率均较低。

## Abstract (en)

**Objective:**

To reassess the predictors for response at 6 months in patients with severe or very severe aplastic anemia（SAA/VSAA）who failed to respond to immunosuppressive therapy（IST）at 3 months.

**Methods:**

We retrospectively analyzed the clinical data of 173 patients with SAA/VSAA from 2017 to 2018 who received IST and were classified as nonresponders at 3 months. Univariate and multivariate logistic regression analysis were used to evaluate factors that could predict the response at 6 months.

**Results:**

Univariate analysis showed that the 3-month hemoglobin（HGB）level（*P*＝0.017）, platelet（PLT）level（*P*＝0.005）, absolute reticulocyte count（ARC）（*P*<0.001）, trough cyclosporine concentration（CsA-C0）（*P*＝0.042）, soluble transferrin receptor（sTfR）level（*P*＝0.003）, improved value of reticulocyte count（ARC^Δ^）（*P*<0.001）, and improved value of soluble transferrin receptor（sTfR^Δ^）level（*P*<0.001）were related to the 6-month response. The results of the multivariate analysis showed that the PLT level（*P*＝0.020）and ARC^Δ^（*P*<0.001）were independent prognostic factors for response at 6 months. If the ARC^Δ^ was less than 6.9×10^9^/L, the 6-month hematological response rate was low, regardless of the patient's PLT count. Survival analysis showed that both the 3-year overall survival（OS）［（80.1±3.9）％ *vs*（97.6±2.6）％, *P*＝0.002］and 3-year event-free survival（EFS）［（31.4±4.5）％ *vs*（86.5±5.3）％, *P*<0.001］of the nonresponders at 6 months were significantly lower than those of the response group.

**Conclusion:**

Residual hematopoietic indicators at 3 months after IST are prognostic parameters. The improved value of the reticulocyte count could reflect whether the bone marrow hematopoiesis is recovering and the degree of recovery. A second treatment could be performed sooner for patients with a very low ARC^Δ^.

免疫抑制治疗（IST）是无同胞相合供者和不适于造血干细胞移植的重型/极重型再生障碍性贫血（SAA/VSAA）患者一线推荐的治疗方案，患者获得血液学反应的中位时间多在开始治疗后2～3个月[1]。若治疗后6个月未脱离血制品输注需行二次治疗，无论是异基因造血干细胞移植（allo-HSCT）或再次IST，均是越早治疗疗效越好[2]。尽管已有不少研究评估预测AA患者IST疗效，但多为初诊时基线评估预测[3]–[5]。且无论使用何种抗人胸腺细胞球蛋白（ATG）或联合血小板生成素（TPO）受体激动剂与否，IST后3个月未获血液学反应AA患者中仅少数会在IST后6个月获得血液学反应[1],[6]–[12]。因此，对IST后3个月无效患者再评估，甄别出IST后6个月仍可能无效的患者尽早进行二次治疗非常重要。本研究回顾性分析173 例IST后3个月无效的SAA患者临床资料，探索影响IST后6个月疗效的相关因素。

## 病例与方法

1. 病例：收集2017年至2018年连续收治的、一线行IST且治疗后3个月未获血液学反应的SAA患者的临床资料和随访信息。在此期间，共331例患者因缺乏同胞全相合供者和（或）年龄大于40岁不适合一线allo-HSCT而接受IST。治疗后3个月疗效评估：12例完全缓解（CR），129例部分缓解（PR），173例未缓解（NR），1例转化为骨髓增生异常综合征（MDS），16例死亡。本研究纳入3个月疗效评估为NR的173例患者。将IST后6个月疗效判定为PR和CR的58例患者计入获得血液学反应组，疗效判定为NR、失访、死亡和行allo-HSCT挽救治疗的患者计入未获血液学反应组，共115例。

2. 诊断和分型：AA诊断参照国际粒细胞减少和AA研究组1987年标准[13]，排除低增生性骨髓增生异常综合征、先天性骨髓造血衰竭、阵发性睡眠性血红蛋白尿症（PNH）等疾病。严重程度分型参照Camitta和Bacigalupo标准[14]–[15]。

3. IST方案：兔ATG（r-ATG）3.0 mg·kg^−1^·d^−1^或猪抗人淋巴细胞球蛋白（p-ALG）20 mg·kg^−1^·d^−1^静脉输注，连用5 d；同时给予泼尼松1 mg·kg^−1^·d^−1^预防即刻输注不良反应和血清病反应。所有患者疾病诊断明确后即开始环孢素A（CsA）口服，以3 mg·kg^−1^·d^−1^分两次口服起始，调整剂量维持CsA谷浓度（CsA-C0）150～250 µg/L、CsA峰浓度（CsA-C2）700～1000 µg/L，达最佳血液学反应并维持至少3个月后开始缓慢减量。

4. 疗效判定与随访：疗效评价采用Camitta 标准[14]，PR及CR均为获得血液学反应。总生存（OS）时间定义为开始IST到死亡或末次随访的时间。无事件生存（EFS）时间定义为开始IST到出现血液学事件或末次随访的时间。血液学事件包括：①死亡；②接受二次IST、环磷酰胺或allo-HSCT；③疾病复发；④疾病转化为克隆性疾病如MDS、急性白血病或溶血性PNH；⑤IST后12个月仍无血液学反应。疾病复发定义为患者达最大疗效（CR/PR）减量或停用免疫抑制剂，因血细胞计数下降需应用二次治疗或血制品输注。患者在CsA减量过程中血细胞计数轻度下降，而加量CsA后血细胞计数可恢复则不列为复发。末次随访时间为2021年4月16日。

5. IST后3个月血液学参数改善值和围IST期发热：IST后3个月血液学参数改善值（X^Δ^，X为血液学参数）定义为同一血液学参数IST后3个月检测值与IST前基线值的差。围IST期发热定义为IST前1个月至IST后3个月期间明确排除其他非感染因素（药物、输血、肿瘤）引起的所有发热事件。发热：单次口腔温度≥38.3 °C（腋温≥38.0 °C）或口腔温度≥38.0 °C（腋温≥37.7 °C）持续超过1 h。发热天数：符合发热诊断标准的自然天数。发热例次：患者从发热开始，经有效治疗直至体温在37.5 °C以下至少维持连续3 d以上，定义为1例次发热。

6. 统计学处理：使用SAS 9.4进行统计学分析。两组之间的分类变量比较采用卡方检验或Fisher精确概率法。定量资料的比较采用Mann-Whitney *U*检验。连续性变量根据临床常用分类或受试者工作曲线（ROC）评估的cutoff值转换为有序分类变量后进行单因素和多因素分析。单因素和多因素分析采用二项Logistic回归。单因素分析有统计学意义的参数纳入多因素分析并进行Spearman相关性分析，相关系数有意义且相关性较强的参数分开纳入多因素回归分析。预测模型的评估采用−2倍对数似然比（−2LL）、决定系数（R^2^）及ROC分析的曲线下面积（AUC）。采用Kaplan-Meier法绘制生存曲线，生存率比较采用Log-rank检验。*P*<0.05为差异具有统计学意义。

## 结果

1. IST后3个月无效患者特征和血液学参数：173例IST后3个月无效SAA/VSAA患者中，男90例，女83例；中位年龄 30（6～72）岁；SAA 90例，VSAA 83例；r-ATG治疗33例，p-ALG治疗140例。IST后6个月共58例（33.5％）获得血液学反应，其中3例CR，55例PR；IST后6个月115例未获血液学反应，其中107例NR、3例失访、1例行allo-HSCT、4例死亡（3例死于感染、1例死于脑出血）。IST后6个月获得与未获血液学反应患者临床特征、血液学参数比较见表1。

**表1:** IST后3个月无效SAA/VSAA患者6个月获得与未获血液学反应组临床特征与血液学参数比较

指标	获得血液学反应（58例）	未获血液学反应（115例）	统计量	*P*值
性别［例（％）］			0.071	0.79
男	31（53.4）	59（51.3）		
女	27（46.6）	56（48.7）		
年龄［岁，*M*（范围）］	24（6~63）	32（7~72）	−1.660	0.097
诊断距IST时间［d，*M*（范围）］	17（6~31）	18（3~49）	−0.991	0.322
疾病严重程度［例（％）］			0.003	0.955
SAA	30（51.7）	60（52.2）		
VSAA	28（48.3）	55（47.8）		
治疗方案［例（％）］			0.190	0.663
r-ATG	10（17.2）	23（20.0）		
p-ALG	48（82.8）	92（80.0）		
G-CSF治疗［例（％）］	50（86.2）	95（82.6）	0.368	0.544
G-CSF治疗有反应［例（％）］^a^	24（48.0）	38（40.0）	0.857	0.355
WBC［×10^9^/L，*M*（IQR）］	2.41（1.87, 3.25）	2.41（1.70, 3.18）	−0.604	0.546
ANC［×10^9^/L，*M*（IQR）］	1.03（0.81, 1.69）	0.90（0.57, 1.51）	−1.514	0.130
ALC［×10^9^/L，*M*（IQR）］	0.95（0.61, 1.39）	1.08（0.57, 1.65）	−0.775	0.438
HGB［g/L，*M*（IQR）］	66（61, 74）	65（59, 73）	−1.742	0.082
PLT［×10^9^/L，*M*（IQR）］	16（12, 24）	13（9, 23）	−2.158	0.031
ARC［×10^9^/L，*M*（IQR）］	43（22, 56）	21（8, 45）	−4.147	<0.001
SF［µg/L，*M*（IQR）］	989（682, 1 552）	1 074（724, 1 818）	−0.781	0.435
sTfR［mg/L，*M*（IQR）］	0.99（0.63, 1.30）	0.72（0.51, 1.01）	−2.908	0.004
CsA-C0［µg/L，*M*（IQR）］	230（177, 288）	188（129, 259）	−2.120	0.034
CsA-C2［µg/L，*M*（IQR）］	650（306, 862）	527（330, 715）	−1.405	0.160
伴PNH克隆［例（％）］	16（27.6）	24（20.9）	0.205	0.650
PNH克隆大小［％，*M*（范围）］	0（0~53）	0（0~61）	−0.248	0.804
染色体核型异常［例（％）］	3（5.2）	13（11.3）	1.727	0.189
淋巴细胞亚群				
CD3^+^T细胞比例［％，*M*（IQR）］	79.0（75.5, 84.2）	79.8（72.1, 85.3）	−0.297	0.767
CD3^+^CD4^+^T细胞比例［％，*M*（IQR）］	35.3（24.4, 47.3）	36.9（25.3, 45.7）	−0.264	0.792
CD3^+^CD8^+^T细胞比例［％，*M*（IQR）］	32.1（26.9, 40.8）	33.2（26.5, 40.4）	−0.264	0.792
Treg比例［％，*M*（IQR）］	1.6（0.8, 2.3）	1.5（0.9, 2.3）	−0.152	0.879
骨髓活检造血面积［例（％）］			2.127	0.145
<10％	12（27.9）	31（41.3）		
≥10％	31（72.1）	44（58.7）		
围IST期发热				
例数［例（％）］	32（55.2）	73（63.5）	1.115	0.291
发热天数［d，*M*（范围）］	1（0~12）	2（0~48）	−1.146	0.252
例次［次，*M*（范围）］	1（0~6）	1（0~8）	−1.299	0.194
随访时间［d，*M*（范围）］	1 084（207~1 484）	1 012（79~1 551）	−1.407	0.159

注：IST：免疫抑制治疗；SAA/VSAA：重型/极重型再生障碍性贫血；r-ATG：兔抗人胸腺细胞球蛋白；p-ALG：猪抗人淋巴细胞球蛋白；ALC：淋巴细胞绝对计数；ARC：网织红细胞绝对计数；SF：血清铁蛋白；sTfR：血清可溶性转铁蛋白受体；CsA-C0：环孢素A血药浓度谷值（用药前）；CsA-C2：环孢素A血药浓度峰值（用药后2 h）；PNH：阵发性睡眠性血红蛋白尿；Treg：调节性T细胞。a：G-CSF治疗10 d后ANC≥0.5×10^9^/L

6个月获得血液学反应组患者IST后3个月的PLT（*P*＝0.031）、ARC（*P*<0.001）、CsA-C0（*P*＝0.034）和 sTfR（*P*＝0.004）水平均明显高于未获血液学反应组。获得血液学反应组患者中位年龄更小（*P*＝0.097），IST后3个月HGB水平更高（*P*＝0.082），但差异无统计学意义。其他方面的差异均无统计学意义。

2. IST后3个月血液学参数改善值比较：IST后6个月获得与未获血液学反应组患者IST后3个月血液学参数改善值比较结果见表2。获得血液学反应组IST后3个月ARC改善值（ARC^Δ^）（*P*<0.001）和sTfR改善值（sTfR^Δ^）（*P*<0.001）水平均明显高于未获血液学反应组。

**表2:** IST后3个月无效SAA/VSAA患者6个月获得与未获血液学反应组血液学参数改善值比较［*M*（*IQR*）］

指标	获得血液学反应（58例）	未获血液学反应组（115例）	*z*值	*P*值
WBC^Δ^（×10^9^/L）	1.00（0.28, 1.84）	0.94（0.25, 1.87）	−0.635	0.525
ANC^Δ^（×10^9^/L）	0.76（0.50, 1.45）	0.68（0.29, 1.36）	−1.559	0.119
ALC^Δ^（×10^9^/L）	0.03（−0.58, 0.29）	0.01（−0.48, 0.36）	−0.577	0.564
HGB^Δ^（g/L）	6（−1, 14）	7（−1, 14）	−0.166	0.868
PLT^Δ^（×10^9^/L）	9（2, 14）	5（0, 13）	−1.211	0.226
ARC^Δ^（×10^9^/L）	33（14, 44）	8（1, 29）	−5.071	<0.001
SF^Δ^（µg/L）	242（0, 939）	483（159, 989）	−1.641	0.101
sTfR^Δ^（mg/L）	0.44（0.14, 0.72）	0.06（−0.10, 0.34）	−3.577	<0.001
CD3^+^T细胞比例^Δ^（％）	2.1（−2.9, 6.0）	3.0（−4.1, 8.2）	−0.323	0.747
CD3^+^CD4^+^T细胞比例^Δ^（％）	−4.6（−11.3, 1.3）	−4.9（−16.2, 1.0）	−0.885	0.376
CD3^+^CD8^+^T细胞比例^Δ^（％）	4.5（1.1, 7.8）	5.0（1.1, 9.6）	−0.677	0.498
Treg比例^Δ^（％）	−0.8（−1.5, −0.1）	−0.7（−1.3, 0.2）	−0.995	0.320

注：IST：免疫抑制治疗；SAA/VSAA：重型/极重型再生障碍性贫血；ALC：淋巴细胞绝对计数；ARC：网织红细胞绝对计数；SF：血清铁蛋白；sTfR：血清可溶性转铁蛋白受体；Treg：调节性T细胞。Δ指IST后3个月检测值与IST前基线值的差

3. IST后6个月血液学反应相关因素分析：将获得与未获血液学反应患者IST后3个月基线参数比较*P*值<0.100的8个参数（年龄、HGB、PLT、ARC、CsA-C0、sTfR、ARC^Δ^、sTfR^Δ^）转换为分类变量后，进行单因素分析。单因素分析显示患者IST后3个月HGB（*P*＝0.017）、PLT（*P*＝0.005）、ARC（*P*<0.001）、CsA-C0（*P*＝0.042）、sTfR（*P*＝0.003）、ARC^Δ^（*P*<0.001）、sTfR^Δ^（*P*<0.001）与IST后6个月疗效有关，纳入多因素分析。而患者的年龄（*P*＝0.103）与IST后6个月疗效无关，不再纳入多因素分析（表3）。

**表3:** IST后3个月未获血液学反应SAA/VSAA患者6个月疗效的单因素分析

因素	IST后6个月有反应［反应例数/总例数（％）］	χ^2^值	*P*值
年龄		2.665	0.103
<40岁	44/117（37.6）		
≥40岁	14/56（25.0）		
HGB		5.668	0.017
<60 g/L	6/35（17.1）		
≥60 g/L	51/129（39.5）		
PLT		7.891	0.005
<10×10^9^/L	5/36（13.9）		
≥10×10^9^/L	52/128（40.6）		
ARC		12.154	<0.001
<20×10^9^/L	11/63（17.5）		
≥20×10^9^/L	45/100（45.0）		
CsA-C0		4.141	0.042
<150 µg/L	9/38（23.7）		
≥150 µg/L	46/108（42.6）		
sTfR		9.267	0.003
<0.86 mg/L	18/70（25.7）		
≥0.86 mg/L	29/55（52.7）		
ARC^Δ^		20.65	<0.001
<6.9×10^9^/L	5/58（8.6）		
≥6.9×10^9^/L	51/105（48.6）		
sTfR^Δ^		13.308	<0.001
<0.165 mg/L	11/57（19.3）		
≥0.165 mg/L	34/65（52.3）		

注：IST：免疫抑制治疗；SAA/VSAA：重型/极重型再生障碍性贫血；ARC：网织红细胞绝对计数；CsA-C0：环孢素A血药浓度谷值（用药前）；sTfR：血清可溶性转铁蛋白受体；ARC^Δ^：网织红细胞绝对计数改善值；sTfR^Δ^：血清可溶性转铁蛋白受体改善值

Spearman相关性分析显示PLT、HGB、CsA-C0的某些相关系数虽有意义，但是相关性不强（*r*_s_<0.300）；而ARC、ARC^Δ^、sTfR和sTfR^Δ^之间相关系数均有意义，且相关性较强（*r*_s_>0.400），见表4。故将ARC、ARC^Δ^、sTfR和sTfR^Δ^分开纳入多因素回归模型，结果见表5。模型1的R^2^及AUC最大，说明模型1的拟合优度及预测效能最高。故我们最终选用模型1来预测IST后6个月疗效。

**表4:** IST后3个月未获血液学反应SAA/VSAA患者6个月疗效相关参数的Spearman相关系数矩阵

	HGB	PLT	ARC	CsA-C0	sTfR	ARC^Δ^	sTfR^Δ^
HGB	1.000	0.263^a^	0.137	−0.012	0.152	0.142	0.174
PLT	0.263^a^	1.000	0.124	0.127	0.171	0.222^a^	0.137
ARC	0.137	0.124	1.000	0.128	0.620^a^	0.621^a^	0.426^a^
CsA-C0	−0.012	0.127	0.128	1.000	0.082	0.032	0.072
sTfR	0.152	0.171	0.620^a^	0.082	1.000	0.493^a^	0.508^a^
ARC^Δ^	0.142	0.222^a^	0.621^a^	0.032	0.493^a^	1.000	0.556^a^
sTfR^Δ^	0.174	0.137	0.426^a^	0.072	0.508^a^	0.556^a^	1.000

注：IST：免疫抑制治疗；SAA/VSAA：重型/极重型再生障碍性贫血；ARC：网织红细胞绝对计数；CsA-C0：环孢素A血药浓度谷值（用药前）；sTfR：血清可溶性转铁蛋白受体；ARC^Δ^：网织红细胞绝对计数改善值；sTfR^Δ^：血清可溶性转铁蛋白受体改善值。^a^*P*<0.01

**表5:** IST后3个月未获血液学反应患者6个月疗效的多因素分析^a^

回归模型	纳入因素	回归方程	−2LL	R^2^	调整R^2^	AUC
模型1	HGB、PLT、CsA-C0和ARC^Δ^	Logit P_有效_＝−3.175+1.281X_1_+2.180X_2_	159.275	0.209	0.284	0.740
模型2	HGB、PLT、CsA-C0和ARC	Logit P_有效_＝−2.533+1.454X_1_+1.247X_3_	172.935	0.131	0.178	0.705
模型3	HGB、PLT、CsA-C0和sTfR^Δ^	Logit P_有效_＝−2.736+1.639X_1_+1.648X_4_	127.469	0.189	0.255	0.717
模型4	HGB、PLT、CsA-C0和sTfR	Logit P_有效_＝−2.210+1.534X_1_+1.127X_5_	139.250	0.132	0.178	0.703

注：a：Logistic回归多因素分析采用stepwise法，当PLT<10×10^9^/L，X_1_＝0，当PLT≥10×10^9^/L，X_1_＝1；当ARC^Δ^<6.9×10^9^/L，X_2_＝0，当ARC^Δ^≥6.9×10^9^/L，X_2_＝1；当ARC<20×10^9^/L，X_3_＝0，当ARC≥20×10^9^/L，X_3_＝1；当sTfR^Δ^<0.165 mg/L，X_4_＝0，当sTfR^Δ^≥0.165 mg/L，X_4_＝1；当sTfR<0.86 mg/L，X_5_＝0，当sTfR≥0.86 mg/L，X_5_＝1。−2LL：−2倍对数似然比；R^2^：决定系数；AUC：曲线下面积

在模型1中，PLT（*P*＝0.020）和ARC^Δ^（*P*<0.001）是IST后6个月疗效的独立预后因素，PLT<10×10^9^/L和ARC^Δ^<6.9×10^9^/L的患者是IST后6个月血液学反应的危险因素。IST后3个月无效患者若PLT<10×10^9^/L且ARC^Δ^<6.9×10^9^/L，IST后6个月血液学反应概率预测值为4％；当PLT≥10×10^9^/L且ARC^Δ^<6.9×10^9^/L，IST后6个月血液学反应概率预测值为13.1％；当PLT<10×10^9^/L且ARC^Δ^≥6.9×10^9^/L，IST后6个月血液学反应概率预测值为27％；当PLT≥10×10^9^/L且ARC^Δ^≥6.9×10^9^/L，IST后6个月血液学反应概率预测值为57.1％。只要ARC^Δ^<6.9×10^9^/L，无论PLT为何值，IST后6个月血液学反应率均较低。

4. 生存分析：获得血液学反应组中位随访时间为1 084（207～1 484）d，未获血液学反应组中位随访时间为1 012（79～1 551）d。至末次随访，IST后6个月未获血液学反应组中22例（19.1％）患者死亡，20例（17.4％）行allo-HSCT，24例（20.9％）NR，32例（27.8％）PR，17例（14.8％）CR。IST后6个月获得血液学反应组中2例（3.5％）患者死亡，具体死因不详；4例（6.9％）患者复发，其中3例再行allo-HSCT，1例行二次免疫抑制治疗后达CR；1例（1.7％）患者转化为溶血性PNH；20例（34.5％）PR；31例（53.4％）CR。IST后6个月未获血液学反应患者3年OS率及EFS率均明显低于获得血液学反应患者［OS：（80.1±3.9）％对（97.6±2.6）％，*P*＝0.002；EFS：（31.4±4.5）％对（86.5±5.3）％，*P*<0.001］，未获血液学反应是影响OS与EFS的危险因素（OS：*HR*＝6.516，95％*CI* 1.512～7.917，*P*＝0.003；EFS：*HR*＝7.723，95％*CI* 4.209～10.360，*P*<0.001）（图1）。

**图1:**
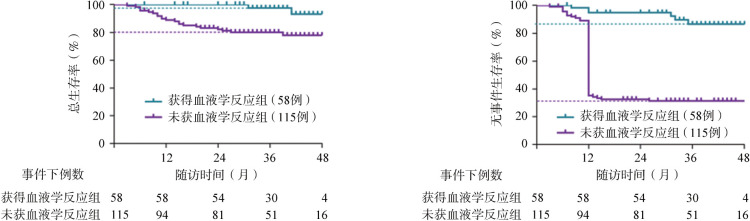
免疫抑制治疗（IST）后6个月获得与未获血液学反应组患者总生存（OS）和无事件生存（EFS）比较

## 讨论

本研究结果显示，对于IST后3个月未获血液学反应的SAA/VSAA患者，继续CsA治疗至IST后6个月仍无效，则发生死亡、持续NR和需要挽救治疗的比例明显高于6个月获得血液学反应患者。且尽管其3年OS率为80.1％，但3年EFS率仅为31.4％，明显低于获得血液学反应组，预后较差。因此，IST后3个月对未获血液学反应的SAA/VSAA患者再评估以预测其IST后6个月恢复自身造血的可能性并加以尽早的治疗策略调整非常重要。

在标准IST治疗前提下，患者年龄、机体状态以及残存造血细胞质量是影响IST后血液学反应获得的最主要影响因素[16]–[17]。研究表明，初治患者基线ARC、PLT、ANC、ALC和sTfR等血液学参数可提示残存造血及预测IST疗效[3],[16],[18]–[19]。本研究发现IST后3个月未获血液学反应患者HGB（*P*＝0.017）、PLT（*P*＝0.005）、ARC（*P*<0.001）、sTfR（*P*＝0.003）与IST后6个月能否获得血液学反应也明显相关。说明IST后3个月无效患者的残存造血指标仍具有明显预后意义。

尽管IST后3个月未达血液学反应标准，但经前期IST治疗血液学参数的改善值可能反映骨髓造血是否正在恢复中以及恢复的程度。本研究表明IST后3个月ARC^Δ^（*P*<0.001）和sTfR^Δ^（*P*<0.001）与IST后6个月疗效有关。血清sTfR在机体不缺铁的状态下，可反映SAA/VSAA患者幼红细胞总量，提示机体骨髓残存造血[20]；而ARC较少受细胞因子治疗和输血的影响，是较为稳定的反映骨髓造血参数。我们认为IST后3个月ARC和sTfR改善增加及增加程度是骨髓恢复造血的明确而客观指标，其与IST后6个月疗效相关的结果与我们前期的推测一致。

单因素分析与IST后6个月疗效相关的因素几乎均为反映残存造血多寡的因素。多因素分析显示模型1的拟合优度及预测效能较好，PLT<10×10^9^/L和ARC^Δ^<6.9×10^9^/L的患者是IST后6个月无效的危险因素。IST后3个月无效患者若PLT<10×10^9^/L且ARC^Δ^<6.9×10^9^/L，则IST后6个月的血液学反应率仅为4％。进一步说明IST后3个月无效患者的血液学参数及其改善值均是提示预后的重要指标。

有文献报道，IST强度不足时不能有效抑制异常免疫，则会导致SAA患者IST失败[21]。Song等[22]的报道也证实了IST后早期CsA血药浓度水平与患者预后相关。本研究也发现对于IST后3个月无效者，CsA-C0高（*P*＝0.034）的患者6个月更可能获得血液学反应。单因素回归分析也显示CsA-C0与IST后6个月疗效有关（*P*＝0.042）。表明免疫抑制治疗强度对IST后6个月疗效的影响，提示IST过程中，规范调整CsA剂量以维持其靶标浓度的重要性。

感染是AA患者IST治疗后主要并发症和死亡原因之一[23]–[24]。本研究显示围IST期有无感染（*P*＝0.291）、发热天数（*P*＝0.252）和发热例次（*P*＝0.194）与IST后6个月疗效无明显相关。这可能与本研究仅纳入IST后3个月无效的活存患者，并未计入早期死亡患者，因而该结果与Atta等[23]和叶蕾等[24]的报告并不矛盾，需谨慎解读。故活存的IST后3个月无效患者6个月能否获得血液学反应主要影响因素仍是疾病本身即造血衰竭程度。在抗生素的及时使用和支持治疗的进步基础上，是否发生感染、感染发生的频次和持续时间虽能从侧面反映AA患者骨髓衰竭程度，但与IST后6个月疗效并无明显相关。

综上，IST后3个月对未获血液学反应的SAA/VSAA患者再评估以预测其6个月疗效非常重要。IST后3个月残存造血仍是影响预后的主要参数。血液学参数改善值可反应骨髓造血是否正在恢复及恢复的程度。本研究表明IST后3个月PLT及ARC改善值是预测6个月疗效的独立预后因素。IST后3个月无效患者若ARC^Δ^<6.9×10^9^/L，无论PLT为何值，IST后6个月的有效率均较低（≤13.1％），可尽早行二次治疗。
